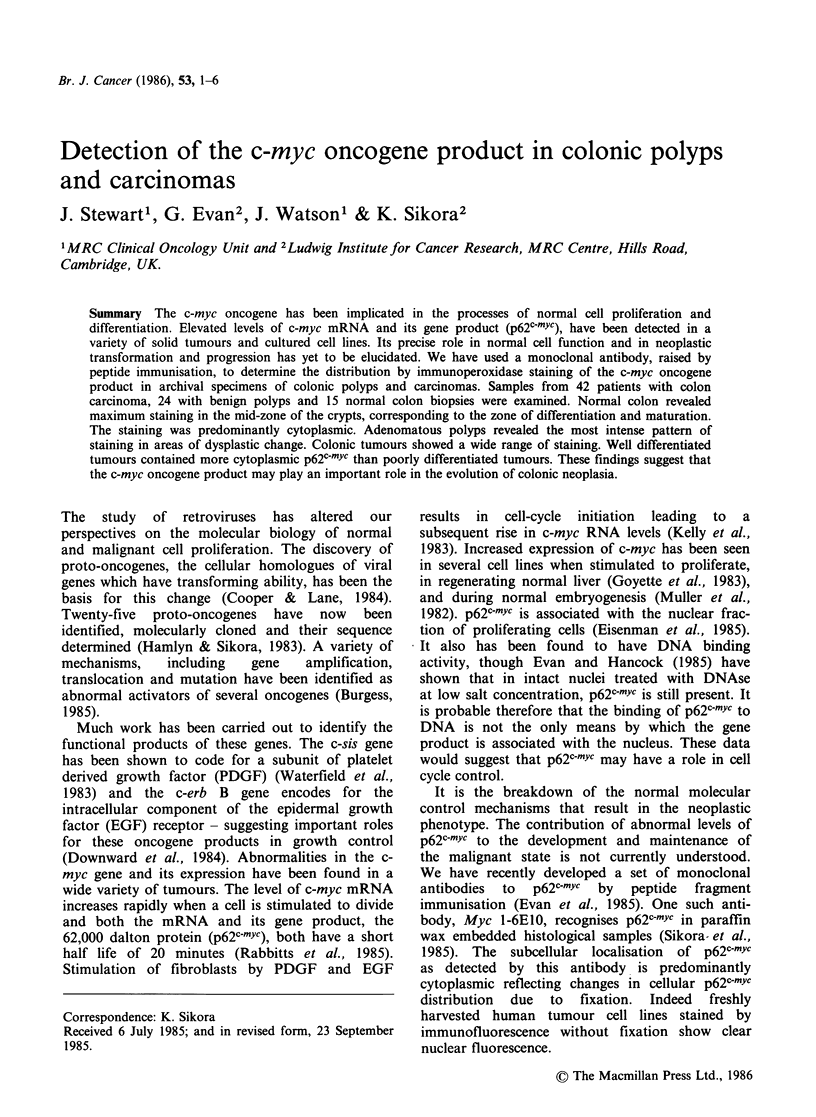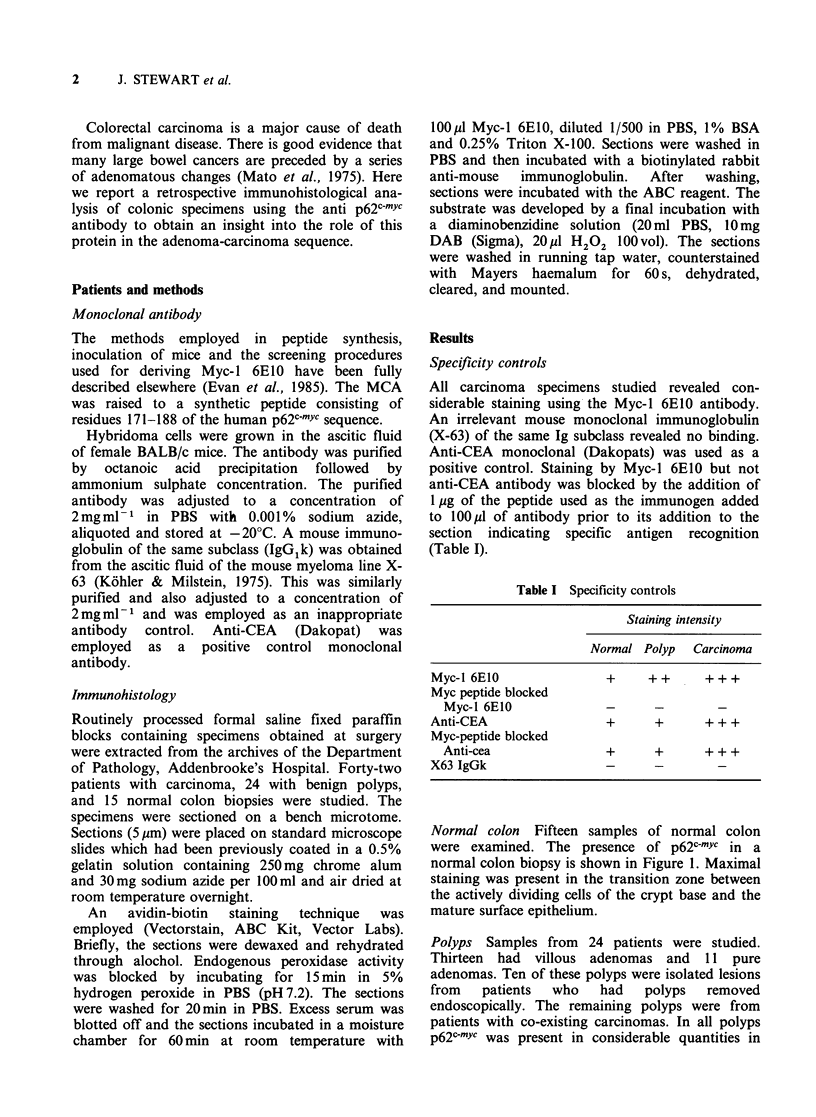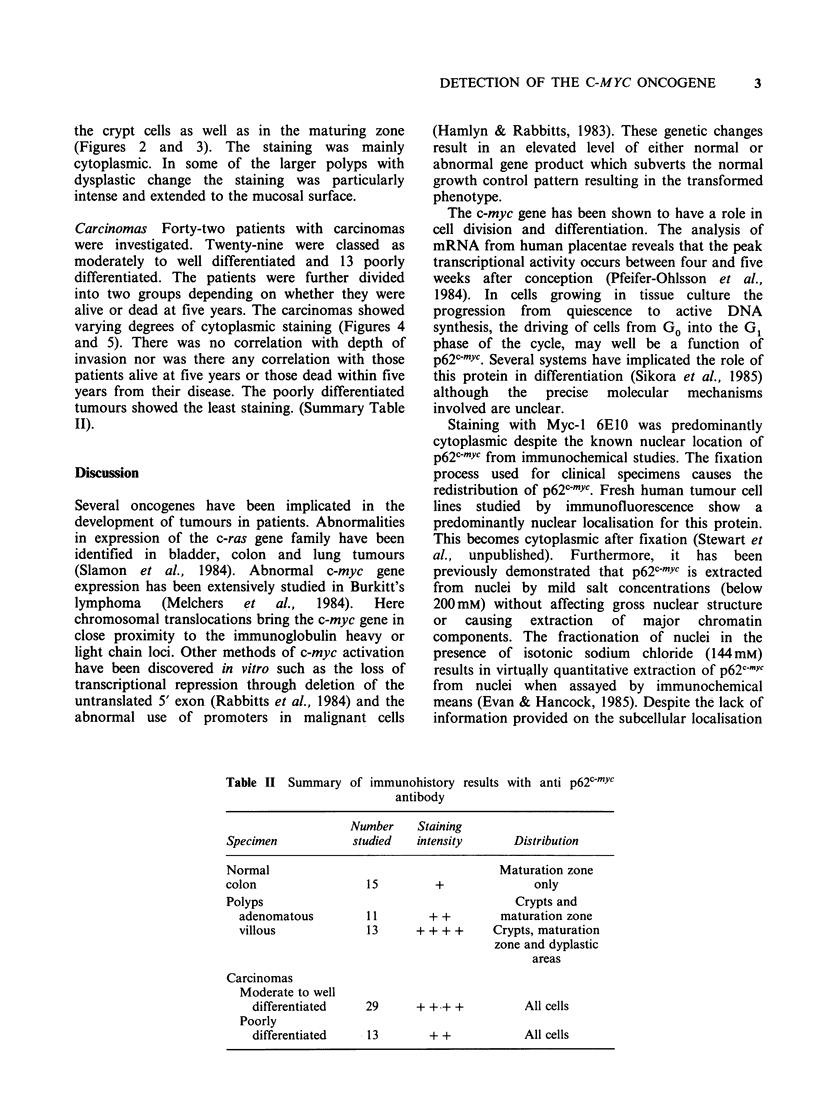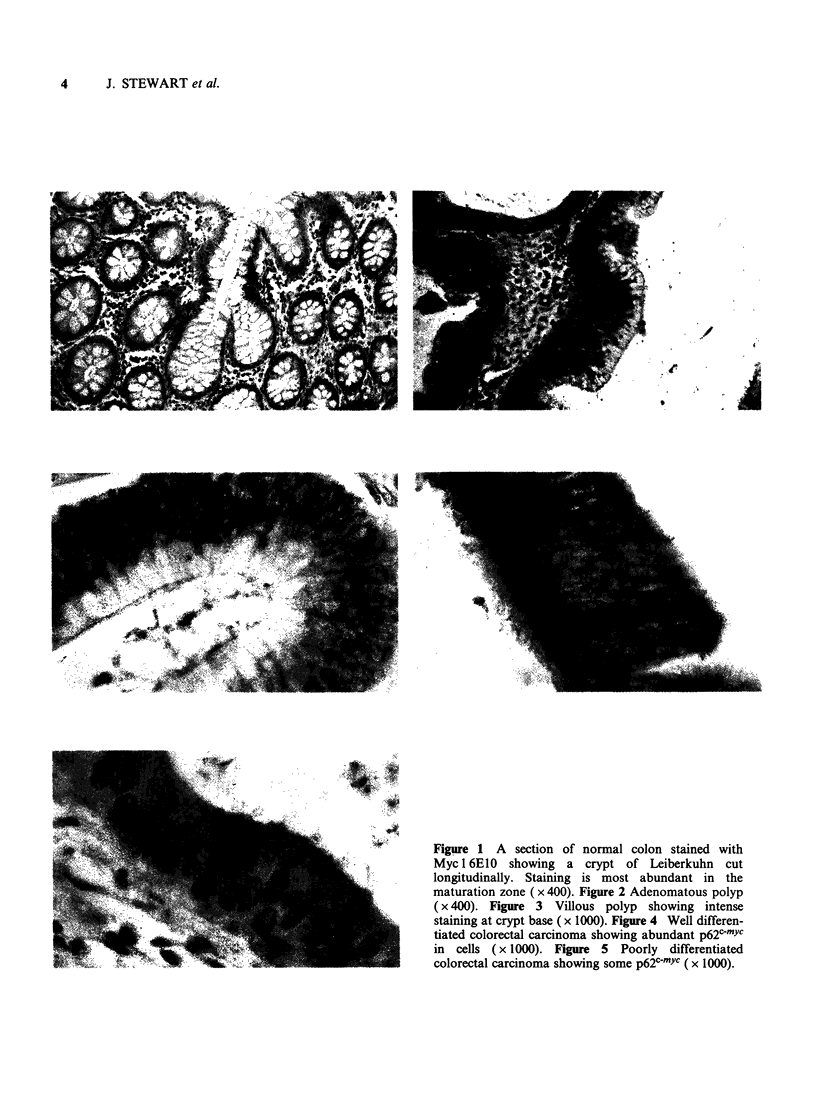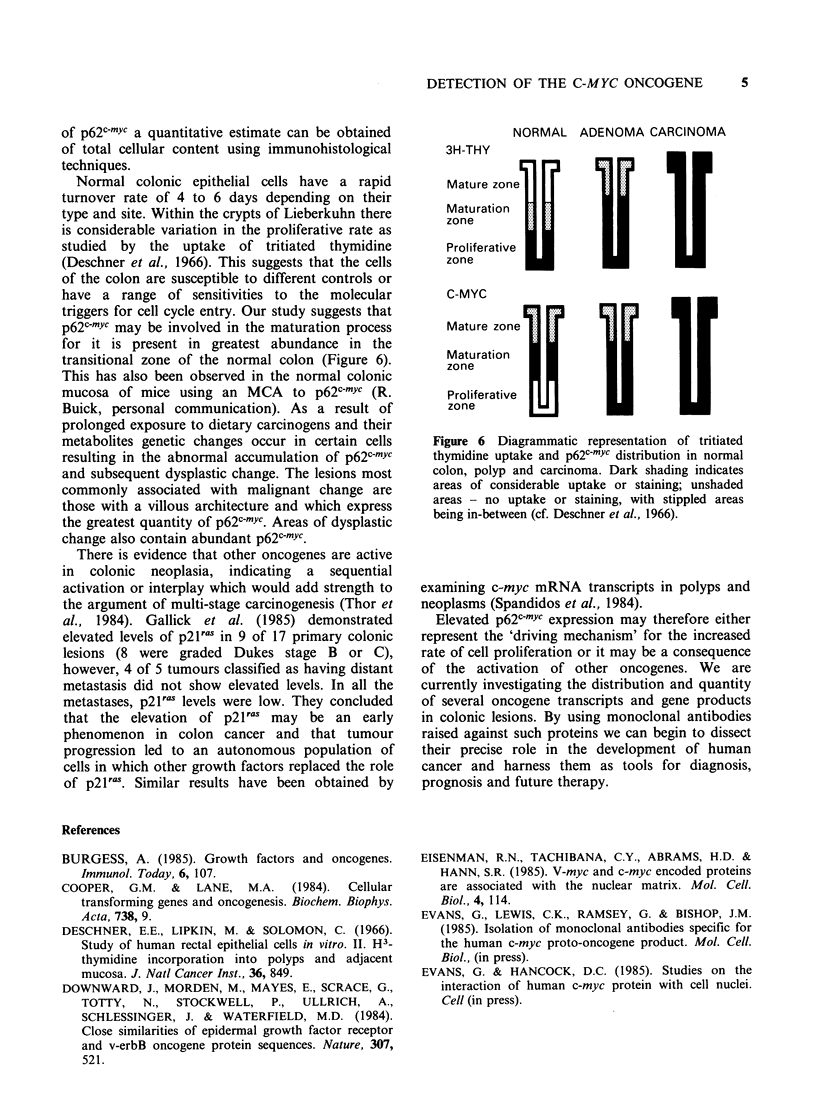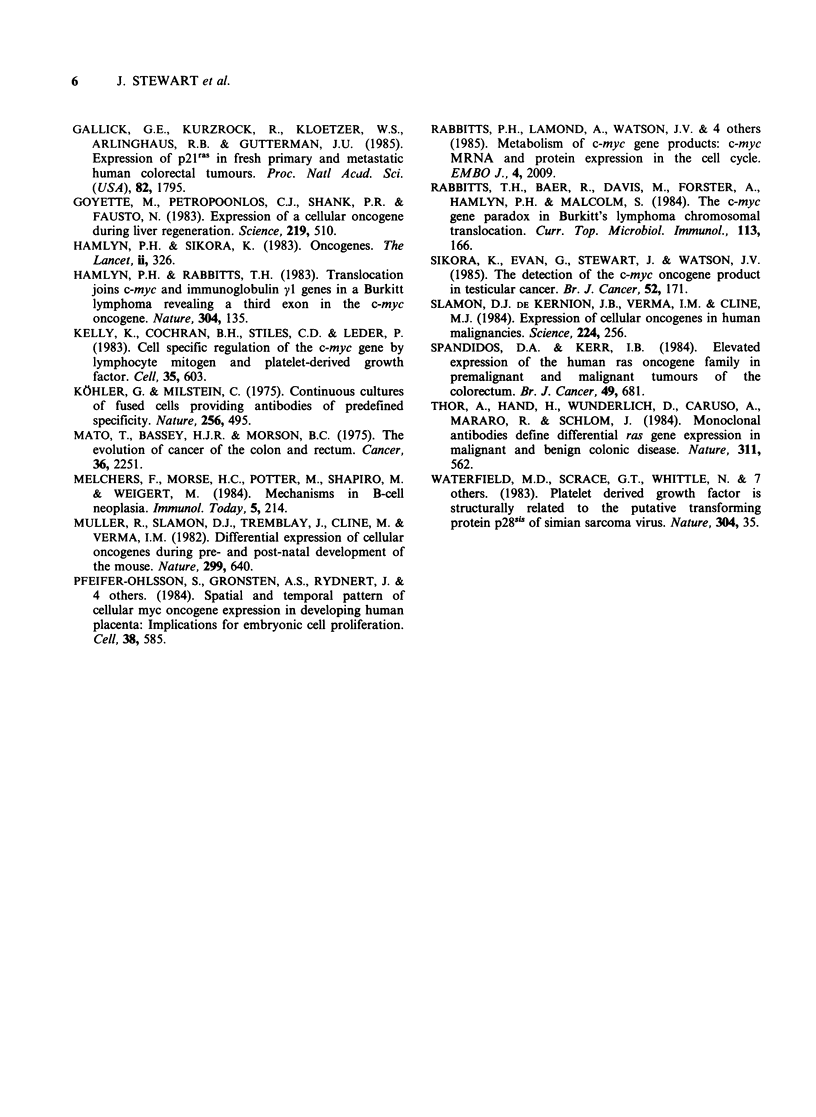# Detection of the c-myc oncogene product in colonic polyps and carcinomas.

**DOI:** 10.1038/bjc.1986.1

**Published:** 1986-01

**Authors:** J. Stewart, G. Evan, J. Watson, K. Sikora

## Abstract

**Images:**


*Br. J. Cancer* (1986), **53**, 1–6

### Detection of the c-*myc* oncogene product in colonic polyps and carcinomas

J. Stewart[1], G. Evan[2], J. Watson[1] & K. Sikora[2]

[1]*MRC Clinical Oncology Unit and* [2]*Ludwig Institute for Cancer Research, MRC Centre, Hills Road, Cambridge, UK.*

**Summary** The c-*myc* oncogene has been implicated in the processes of normal cell proliferation and differentiation. Elevated levels of c-*myc* mRNA and its gene product ($p62^{c\text{-}myc}$), have been detected in a variety of solid tumours and cultured cell lines. Its precise role in normal cell function and in neoplastic transformation and progression has yet to be elucidated. We have used a monoclonal antibody, raised by peptide immunisation, to determine the distribution by immunoperoxidase staining of the c-*myc* oncogene product in archival specimens of colonic polyps and carcinomas. Samples from 42 patients with colon carcinoma, 24 with benign polyps and 15 normal colon biopsies were examined. Normal colon revealed maximum staining in the mid-zone of the crypts, corresponding to the zone of differentiation and maturation. The staining was predominantly cytoplasmic. Adenomatous polyps revealed the most intense pattern of staining in areas of dysplastic change. Colonic tumours showed a wide range of staining. Well differentiated tumours contained more cytoplasmic $p62^{c\text{-}myc}$ than poorly differentiated tumours. These findings suggest that the c-*myc* oncogene product may play an important role in the evolution of colonic neoplasia.

The study of retroviruses has altered our perspectives on the molecular biology of normal and malignant cell proliferation. The discovery of proto-oncogenes, the cellular homologues of viral genes which have transforming ability, has been the basis for this change (Cooper & Lane, 1984). Twenty-five proto-oncogenes have now been identified, molecularly cloned and their sequence determined (Hamlyn & Sikora, 1983). A variety of mechanisms, including gene amplification, translocation and mutation have been identified as abnormal activators of several oncogenes (Burgess, 1985).

Much work has been carried out to identify the functional products of these genes. The c-*sis* gene has been shown to code for a subunit of platelet derived growth factor (PDGF) (Waterfield *et al.*, 1983) and the c-*erb* B gene encodes for the intracellular component of the epidermal growth factor (EGF) receptor – suggesting important roles for these oncogene products in growth control (Downward *et al.*, 1984). Abnormalities in the c-*myc* gene and its expression have been found in a wide variety of tumours. The level of c-*myc* mRNA increases rapidly when a cell is stimulated to divide and both the mRNA and its gene product, the 62,000 dalton protein ($p62^{c\text{-}myc}$), both have a short half life of 20 minutes (Rabbitts *et al.*, 1985). Stimulation of fibroblasts by PDGF and EGF results in cell-cycle initiation leading to a subsequent rise in c-*myc* RNA levels (Kelly *et al.*, 1983). Increased expression of c-*myc* has been seen in several cell lines when stimulated to proliferate, in regenerating normal liver (Goyette *et al.*, 1983), and during normal embryogenesis (Muller *et al.*, 1982). $p62^{c\text{-}myc}$ is associated with the nuclear fraction of proliferating cells (Eisenman *et al.*, 1985). It also has been found to have DNA binding activity, though Evan and Hancock (1985) have shown that in intact nuclei treated with DNAse at low salt concentration, $p62^{c\text{-}myc}$ is still present. It is probable therefore that the binding of $p62^{c\text{-}myc}$ to DNA is not the only means by which the gene product is associated with the nucleus. These data would suggest that $p62^{c\text{-}myc}$ may have a role in cell cycle control.

It is the breakdown of the normal molecular control mechanisms that result in the neoplastic phenotype. The contribution of abnormal levels of $p62^{c\text{-}myc}$ to the development and maintenance of the malignant state is not currently understood. We have recently developed a set of monoclonal antibodies to $p62^{c\text{-}myc}$ by peptide fragment immunisation (Evan *et al.*, 1985). One such antibody, *Myc* 1-6E10, recognises $p62^{c\text{-}myc}$ in paraffin wax embedded histological samples (Sikora *et al.*, 1985). The subcellular localisation of $p62^{c\text{-}myc}$ as detected by this antibody is predominantly cytoplasmic reflecting changes in cellular $p62^{c\text{-}myc}$ distribution due to fixation. Indeed freshly harvested human tumour cell lines stained by immunofluorescence without fixation show clear nuclear fluorescence.

Correspondence: K. Sikora
Received 6 July 1985; and in revised form, 23 September 1985.

© The Macmillan Press Ltd., 1986

Colorectal carcinoma is a major cause of death from malignant disease. There is good evidence that many large bowel cancers are preceded by a series of adenomatous changes (Mato *et al.*, 1975). Here we report a retrospective immunohistological analysis of colonic specimens using the anti $p62^{c\text{-}myc}$ antibody to obtain an insight into the role of this protein in the adenoma-carcinoma sequence.

### Patients and methods

### *Monoclonal antibody*

The methods employed in peptide synthesis, inoculation of mice and the screening procedures used for deriving Myc-1 6E10 have been fully described elsewhere (Evan *et al.*, 1985). The MCA was raised to a synthetic peptide consisting of residues 171–188 of the human $p62^{c\text{-}myc}$ sequence.

Hybridoma cells were grown in the ascitic fluid of female BALB/c mice. The antibody was purified by octanoic acid precipitation followed by ammonium sulphate concentration. The purified antibody was adjusted to a concentration of $2\,mg\,ml^{-1}$ in PBS with 0.001% sodium azide, aliquoted and stored at $-20°C$. A mouse immunoglobulin of the same subclass ($IgG_1k$) was obtained from the ascitic fluid of the mouse myeloma line X-63 (Köhler & Milstein, 1975). This was similarly purified and also adjusted to a concentration of $2\,mg\,ml^{-1}$ and was employed as an inappropriate antibody control. Anti-CEA (Dakopat) was employed as a positive control monoclonal antibody.

### *Immunohistology*

Routinely processed formal saline fixed paraffin blocks containing specimens obtained at surgery were extracted from the archives of the Department of Pathology, Addenbrooke's Hospital. Forty-two patients with carcinoma, 24 with benign polyps, and 15 normal colon biopsies were studied. The specimens were sectioned on a bench microtome. Sections ($5\,\mu m$) were placed on standard microscope slides which had been previously coated in a 0.5% gelatin solution containing 250 mg chrome alum and 30 mg sodium azide per 100 ml and air dried at room temperature overnight.

An avidin-biotin staining technique was employed (Vectorstain, ABC Kit, Vector Labs). Briefly, the sections were dewaxed and rehydrated through alochol. Endogenous peroxidase activity was blocked by incubating for 15 min in 5% hydrogen peroxide in PBS (pH 7.2). The sections were washed for 20 min in PBS. Excess serum was blotted off and the sections incubated in a moisture chamber for 60 min at room temperature with $100\,\mu l$ Myc-1 6E10, diluted 1/500 in PBS, 1% BSA and 0.25% Triton X-100. Sections were washed in PBS and then incubated with a biotinylated rabbit anti-mouse immunoglobulin. After washing, sections were incubated with the ABC reagent. The substrate was developed by a final incubation with a diaminobenzidine solution (20 ml PBS, 10 mg DAB (Sigma), $20\,\mu l$ $H_2O_2$ 100 vol). The sections were washed in running tap water, counterstained with Mayers haemalum for 60 s, dehydrated, cleared, and mounted.

### Results

### *Specificity controls*

All carcinoma specimens studied revealed considerable staining using the Myc-1 6E10 antibody. An irrelevant mouse monoclonal immunoglobulin (X-63) of the same Ig subclass revealed no binding. Anti-CEA monoclonal (Dakopats) was used as a positive control. Staining by Myc-1 6E10 but not anti-CEA antibody was blocked by the addition of $1\,\mu g$ of the peptide used as the immunogen added to $100\,\mu l$ of antibody prior to its addition to the section indicating specific antigen recognition (Table I).

**Table I** Specificity controls

| | *Staining intensity* | | |
|---|---|---|---|
| | *Normal* | *Polyp* | *Carcinoma* |
| Myc-1 6E10 | + | ++ | +++ |
| Myc peptide blocked Myc-1 6E10 | − | − | − |
| Anti-CEA | + | + | +++ |
| Myc-peptide blocked Anti-cea | + | + | +++ |
| X63 IgGk | − | − | − |

*Normal colon* Fifteen samples of normal colon were examined. The presence of $p62^{c\text{-}myc}$ in a normal colon biopsy is shown in Figure 1. Maximal staining was present in the transition zone between the actively dividing cells of the crypt base and the mature surface epithelium.

*Polyps* Samples from 24 patients were studied. Thirteen had villous adenomas and 11 pure adenomas. Ten of these polyps were isolated lesions from patients who had polyps removed endoscopically. The remaining polyps were from patients with co-existing carcinomas. In all polyps $p62^{c\text{-}myc}$ was present in considerable quantities in the crypt cells as well as in the maturing zone (Figures 2 and 3). The staining was mainly cytoplasmic. In some of the larger polyps with dysplastic change the staining was particularly intense and extended to the mucosal surface.

*Carcinomas* Forty-two patients with carcinomas were investigated. Twenty-nine were classed as moderately to well differentiated and 13 poorly differentiated. The patients were further divided into two groups depending on whether they were alive or dead at five years. The carcinomas showed varying degrees of cytoplasmic staining (Figures 4 and 5). There was no correlation with depth of invasion nor was there any correlation with those patients alive at five years or those dead within five years from their disease. The poorly differentiated tumours showed the least staining. (Summary Table II).

### Discussion

Several oncogenes have been implicated in the development of tumours in patients. Abnormalities in expression of the c-*ras* gene family have been identified in bladder, colon and lung tumours (Slamon *et al.*, 1984). Abnormal c-*myc* gene expression has been extensively studied in Burkitt's lymphoma (Melchers *et al.*, 1984). Here chromosomal translocations bring the c-*myc* gene in close proximity to the immunoglobulin heavy or light chain loci. Other methods of c-*myc* activation have been discovered *in vitro* such as the loss of transcriptional repression through deletion of the untranslated 5′ exon (Rabbitts *et al.*, 1984) and the abnormal use of promoters in malignant cells (Hamlyn & Rabbitts, 1983). These genetic changes result in an elevated level of either normal or abnormal gene product which subverts the normal growth control pattern resulting in the transformed phenotype.

The c-*myc* gene has been shown to have a role in cell division and differentiation. The analysis of mRNA from human placentae reveals that the peak transcriptional activity occurs between four and five weeks after conception (Pfeifer-Ohlsson *et al.*, 1984). In cells growing in tissue culture the progression from quiescence to active DNA synthesis, the driving of cells from $G_0$ into the $G_1$ phase of the cycle, may well be a function of $p62^{c\text{-}myc}$. Several systems have implicated the role of this protein in differentiation (Sikora *et al.*, 1985) although the precise molecular mechanisms involved are unclear.

Staining with Myc-1 6E10 was predominantly cytoplasmic despite the known nuclear location of $p62^{c\text{-}myc}$ from immunochemical studies. The fixation process used for clinical specimens causes the redistribution of $p62^{c\text{-}myc}$. Fresh human tumour cell lines studied by immunofluorescence show a predominantly nuclear localisation for this protein. This becomes cytoplasmic after fixation (Stewart *et al.*, unpublished). Furthermore, it has been previously demonstrated that $p62^{c\text{-}myc}$ is extracted from nuclei by mild salt concentrations (below 200 mM) without affecting gross nuclear structure or causing extraction of major chromatin components. The fractionation of nuclei in the presence of isotonic sodium chloride (144 mM) results in virtually quantitative extraction of $p62^{c\text{-}myc}$ from nuclei when assayed by immunochemical means (Evan & Hancock, 1985). Despite the lack of information provided on the subcellular localisation

**Table II** Summary of immunohistory results with anti $p62^{c\text{-}myc}$ antibody

| *Specimen* | *Number studied* | *Staining intensity* | *Distribution* |
|---|---|---|---|
| Normal colon | 15 | + | Maturation zone only |
| Polyps | | | |
| adenomatous | 11 | ++ | Crypts and maturation zone |
| villous | 13 | ++++ | Crypts, maturation zone and dyplastic areas |
| Carcinomas | | | |
| Moderate to well differentiated | 29 | ++++ | All cells |
| Poorly differentiated | 13 | ++ | All cells |

**Figure 1** A section of normal colon stained with Myc1 6E10 showing a crypt of Leiberkuhn cut longitudinally. Staining is most abundant in the maturation zone ( × 400). **Figure 2** Adenomatous polyp ( × 400). **Figure 3** Villous polyp showing intense staining at crypt base ( × 1000). **Figure 4** Well differentiated colorectal carcinoma showing abundant $p62^{c\text{-}myc}$ in cells ( × 1000). **Figure 5** Poorly differentiated colorectal carcinoma showing some $p62^{c\text{-}myc}$ ( × 1000).

of p62$^{c\text{-}myc}$ a quantitative estimate can be obtained of total cellular content using immunohistological techniques.

Normal colonic epithelial cells have a rapid turnover rate of 4 to 6 days depending on their type and site. Within the crypts of Lieberkuhn there is considerable variation in the proliferative rate as studied by the uptake of tritiated thymidine (Deschner *et al.*, 1966). This suggests that the cells of the colon are susceptible to different controls or have a range of sensitivities to the molecular triggers for cell cycle entry. Our study suggests that p62$^{c\text{-}myc}$ may be involved in the maturation process for it is present in greatest abundance in the transitional zone of the normal colon (Figure 6). This has also been observed in the normal colonic mucosa of mice using an MCA to p62$^{c\text{-}myc}$ (R. Buick, personal communication). As a result of prolonged exposure to dietary carcinogens and their metabolites genetic changes occur in certain cells resulting in the abnormal accumulation of p62$^{c\text{-}myc}$ and subsequent dysplastic change. The lesions most commonly associated with malignant change are those with a villous architecture and which express the greatest quantity of p62$^{c\text{-}myc}$. Areas of dysplastic change also contain abundant p62$^{c\text{-}myc}$.

There is evidence that other oncogenes are active in colonic neoplasia, indicating a sequential activation or interplay which would add strength to the argument of multi-stage carcinogenesis (Thor *et al.*, 1984). Gallick *et al.* (1985) demonstrated elevated levels of p21$^{ras}$ in 9 of 17 primary colonic lesions (8 were graded Dukes stage B or C), however, 4 of 5 tumours classified as having distant metastasis did not show elevated levels. In all the metastases, p21$^{ras}$ levels were low. They concluded that the elevation of p21$^{ras}$ may be an early phenomenon in colon cancer and that tumour progression led to an autonomous population of cells in which other growth factors replaced the role of p21$^{ras}$. Similar results have been obtained by examining c-*myc* mRNA transcripts in polyps and neoplasms (Spandidos *et al.*, 1984).

**Figure 6** Diagrammatic representation of tritiated thymidine uptake and p62$^{c\text{-}myc}$ distribution in normal colon, polyp and carcinoma. Dark shading indicates areas of considerable uptake or staining; unshaded areas – no uptake or staining, with stippled areas being in-between (cf. Deschner *et al.*, 1966).

Elevated p62$^{c\text{-}myc}$ expression may therefore either represent the 'driving mechanism' for the increased rate of cell proliferation or it may be a consequence of the activation of other oncogenes. We are currently investigating the distribution and quantity of several oncogene transcripts and gene products in colonic lesions. By using monoclonal antibodies raised against such proteins we can begin to dissect their precise role in the development of human cancer and harness them as tools for diagnosis, prognosis and future therapy.

### References

BURGESS, A. (1985). Growth factors and oncogenes. *Immunol. Today*, **6**, 107.

COOPER, G.M. & LANE, M.A. (1984). Cellular transforming genes and oncogenesis. *Biochem. Biophys. Acta*, **738**, 9.

DESCHNER, E.E., LIPKIN, M. & SOLOMON, C. (1966). Study of human rectal epithelial cells *in vitro*. II. H$^3$-thymidine incorporation into polyps and adjacent mucosa. *J. Natl Cancer Inst.*, **36**, 849.

DOWNWARD, J., MORDEN, M., MAYES, E., SCRACE, G., TOTTY, N., STOCKWELL, P., ULLRICH, A., SCHLESSINGER, J. & WATERFIELD, M.D. (1984). Close similarities of epidermal growth factor receptor and v-erbB oncogene protein sequences. *Nature*, **307**, 521.

EISENMAN, R.N., TACHIBANA, C.Y., ABRAMS, H.D. & HANN, S.R. (1985). V-*myc* and c-*myc* encoded proteins are associated with the nuclear matrix. *Mol. Cell. Biol.*, **4**, 114.

EVANS, G., LEWIS, C.K., RAMSEY, G. & BISHOP, J.M. (1985). Isolation of monoclonal antibodies specific for the human c-*myc* proto-oncogene product. *Mol. Cell. Biol.*, (in press).

EVANS, G. & HANCOCK, D.C. (1985). Studies on the interaction of human c-*myc* protein with cell nuclei. *Cell* (in press).

GALLICK, G.E., KURZROCK, R., KLOETZER, W.S., ARLINGHAUS, R.B. & GUTTERMAN, J.U. (1985). Expression of p21$^{ras}$ in fresh primary and metastatic human colorectal tumours. *Proc. Natl Acad. Sci. (USA)*, **82,** 1795.

GOYETTE, M., PETROPOONLOS, C.J., SHANK, P.R. & FAUSTO, N. (1983). Expression of a cellular oncogene during liver regeneration. *Science,* **219,** 510.

HAMLYN, P.H. & SIKORA, K. (1983). Oncogenes. *The Lancet,* **ii,** 326.

HAMLYN, P.H. & RABBITTS, T.H. (1983). Translocation joins c-*myc* and immunoglobulin $\gamma 1$ genes in a Burkitt lymphoma revealing a third exon in the c-*myc* oncogene. *Nature,* **304,** 135.

KELLY, K., COCHRAN, B.H., STILES, C.D. & LEDER, P. (1983). Cell specific regulation of the c-*myc* gene by lymphocyte mitogen and platelet-derived growth factor. *Cell,* **35,** 603.

KÖHLER, G. & MILSTEIN, C. (1975). Continuous cultures of fused cells providing antibodies of predefined specificity. *Nature,* **256,** 495.

MATO, T., BASSEY, H.J.R. & MORSON, B.C. (1975). The evolution of cancer of the colon and rectum. *Cancer,* **36,** 2251.

MELCHERS, F., MORSE, H.C., POTTER, M., SHAPIRO, M. & WEIGERT, M. (1984). Mechanisms in B-cell neoplasia. *Immunol. Today,* **5,** 214.

MULLER, R., SLAMON, D.J., TREMBLAY, J., CLINE, M. & VERMA, I.M. (1982). Differential expression of cellular oncogenes during pre- and post-natal development of the mouse. *Nature,* **299,** 640.

PFEIFER-OHLSSON, S., GRONSTEN, A.S., RYDNERT, J. & 4 others. (1984). Spatial and temporal pattern of cellular myc oncogene expression in developing human placenta: Implications for embryonic cell proliferation. *Cell,* **38,** 585.

RABBITTS, P.H., LAMOND, A., WATSON, J.V. & 4 others (1985). Metabolism of c-*myc* gene products: c-*myc* MRNA and protein expression in the cell cycle. *EMBO J.,* **4,** 2009.

RABBITTS, T.H., BAER, R., DAVIS, M., FORSTER, A., HAMLYN, P.H. & MALCOLM, S. (1984). The c-*myc* gene paradox in Burkitt's lymphoma chromosomal translocation. *Curr. Top. Microbiol. Immunol.,* **113,** 166.

SIKORA, K., EVAN, G., STEWART, J. & WATSON, J.V. (1985). The detection of the c-*myc* oncogene product in testicular cancer. *Br. J. Cancer,* **52,** 171.

SLAMON, D.J. DE KERNION, J.B., VERMA, I.M. & CLINE, M.J. (1984). Expression of cellular oncogenes in human malignancies. *Science,* **224,** 256.

SPANDIDOS, D.A. & KERR, I.B. (1984). Elevated expression of the human ras oncogene family in premalignant and malignant tumours of the colorectum. *Br. J. Cancer,* **49,** 681.

THOR, A., HAND, H., WUNDERLICH, D., CARUSO, A., MARARO, R. & SCHLOM, J. (1984). Monoclonal antibodies define differential *ras* gene expression in malignant and benign colonic disease. *Nature,* **311,** 562.

WATERFIELD, M.D., SCRACE, G.T., WHITTLE, N. & 7 others. (1983). Platelet derived growth factor is structurally related to the putative transforming protein p28$^{sis}$ of simian sarcoma virus. *Nature,* **304,** 35.